# The Ethics of Rationing of Critical Care Services: Should Technology Assessment Play a Role?

**DOI:** 10.1155/2009/915197

**Published:** 2009-11-10

**Authors:** Eric L. Bloomfield

**Affiliations:** Department of Anesthesiology, Mayo Clinic, 200 First Street SW, Rochester, MN 55905, USA

## Abstract

The costs of health care continue to increase rapidly and steeply in the United States. One area of great expense is that of intensive care units (ICUs). The causes of inflation have not been addressed effectively. ICU resources could become stretched such that they may no longer be available. This paper discusses some of the ethics and concerns behind decision making when providing ICU services in the United States. In particular, the use of electronic records with decision making tools, risk-analysis methods, and documentation of patient wishes for extraordinary care may help with better utilization of resources in the future.

## 1. The Financial Problem in Health Care in the United States

Since the advent of Medicare in this country, health care costs have been increasing. In 1970, the United States and Canada spent similar amounts on health care, at around $100 per capita [1]. As of 2000, this figure has increased to more than $4,500 per capita in the United States, with spending on health services about 13% of the gross national product [1], much higher than in the rest of the world. It is estimated that more than 1% of the gross domestic product is spent on intensive care unit (ICU) services [2].

What does the American public receive for the amount of money spent on health care? For one, life expectancy in the United States is not as long as in other developed countries such as Japan that spend much less on health care [3]. Also, large discrepancies in life expectancy exist among different racial groups. White females in the United States live to be about 78.8 years, whereas African American males have a life expectancy of around 64 years of age [3]. Why is this?

Newhouse [5] describes 5 factors associated with increased health care expenditures: increased insurance usage, aging, increases in income, increases in supplier-induced demand, and other miscellaneous factors that exist in the service sector. The question sometimes arises whether this problem could be solved by deregulated economic competition. President Clinton in the 90s proposed that “managed competition” would bring costs under control. But the price of health care continues to increase, the demand continues to increase, the resources are starting to decrease, the baby boomers will be reaching age 65 years by 2011, and now approximately 46 million people in the country do not have health care coverage [6]. It is a problem that continues to grow.

## 2. Economics of Health Care in the United States

Supply and demand forces have always played a role in economics. If supply is low and demand is high, then prices will naturally rise. This also works in reverse. Generalized competition in the free marketplace has been known to control the price of goods and services. With generalized oversupply, the prices do tend to go down [7].

Free market competition should hold down managed care prices so that marginal costs would equal marginal revenues for most goods and services. However, certain factors must be met according to Arrow [4]. (1) There are sufficient buyers and sellers of each economic good such that no single player has any power over the price. (2) The good is homogeneous; all producers produce exactly the same good so that the market cannot be segmented on the basis of different goods. (3) There is perfect information; all buyers and sellers have reliable information on all relevant variables, such as prices and quantities. (4) There are no barriers to entry and exit as producers start producing items on terms that are equivalent to those already in the industry. And (5) the good must not produce any secondary benefits or costs to others (Box 1).

The question is “Does health care meet all of those criteria?” The answer is clearly no. In particular, free access to health care is not possible because no form of universal health insurance is available. This hinders access to health care for millions of Americans. This, in particular, plays a huge role in the administration of critical care services, which is some of the most expensive care provided today [1].

Health care inflation is caused by 3 factors (Box 2). One of them is asymmetry of information [8]. It is defined by Folland et al. [8] as “situations in which the parties on the opposite side of a transaction have differing amounts of relevant information.” Health care, however, manifests with many information asymmetries [9]. Patients often do not have perfect information regarding their choices in health care. Physicians may lack perfect information that they need. In essence, they may spend needless dollars without achieving optimal outcomes. It is hoped that electronic systems could make it easier for patients to understand their choices. The American Medical Association is making a better effort for patients to connect with their physicians [10].

The second factor relates to health insurance itself. The 60s and 70s saw a rapid increase in health care services. Most people argue that this was a result of the “fee for service” model. “Moral Hazard,” as defined by Folland et al. [8], expresses the additional quantity of health care resulting from a decrease in the net price of care attributable to insurance. This was borne out in the RAND health insurance experiment [5]. People tended to use more health care resources when they had to pay less copay. This was never shown in the ICU setting, but it lends credibility to the idea that more health care resources will probably be used if they are free.

The last factor involved in health care inflation is technology. Technology has had an expanding role, including in the consideration of new drugs, devices, processes, and different types of new procedures. An example of this is the automatic internal cardiac defibrillator placed in patients with intrinsic heart dysrhythmias or sudden death syndrome. This is a costly device that is useful in improving outcomes [11]. However, it can be shown to not be cost-effective when placed in certain patients with severe congestive heart failure [12]. Cost-effectiveness of technology devices has been defined as less than $50,000 per quality-adjusted life year saved [13]. However, most providers do not act on data such as this in medical decision making. Technology is probably still regarded as the forefront of health care, but it can lead to rapidly increasing health care costs.

Weisbrod [9] described technology at 3 different levels depending on what it can achieve. He describes it as either “nontechnology,” “half-way,” or “high” technology depending on what it achieves and the costs involved. Likewise, Scitovsky [14] has suggested that technology has increased costs in many ways. Technologies can increase the quality of care but at the same time increase the overall cost, which thus limits the maximal value derived from the technology. This requires assessment.

Although technology has provided changes in human physiology and outcomes, as described by Fogel and Costa [15], it also has sometimes led to decreasing returns. Kindig [3] describes in his book, “Purchasing Population Health: Paying for Results,” that there is a limit to the amount of money that can be put into health care to obtain an optimal level of results. Beyond that limit, Kindig states that there are decreasing returns in health care outcomes. This could be in turn attributed to iatrogenic complications and money wasted on ineffective or inefficient technology. Kindig [3] believes that achieving a healthier population will require more than just health care; it also will involve concepts such as social justice and better educational systems.

Of all of these factors, technology probably plays the strongest role in health care inflation. With proper technology assessment of health care resources, would rationing be necessary for critical care services?

## 3. Technology Assessment

Technology assessment can be broad; it involves looking at the costs of all items, such as diagnostic devices, therapeutic devices, medications, information systems, and procedures [16]. Unfortunately, proper modes of assessment are not often used for new modes of technology. In particular, information systems that are touted as the new wave to make health care more efficient have received little assessment in the way of cost-effectiveness analysis. Countries such as New Zealand have done a much better job than the United States of analyzing technology before implementation [17].

Differences exist in the manner in which clinical coverage is provided in ICUs in the United States [18]. More than half of the ICUs in the United States do not have a director [19]. Care of patients in these ICUs is managed by many different physicians. Evidence has been accumulating that ICU patients whose care is managed by an intensivist tend to have better outcomes [20], which tends to coincide with better quality control. One of W. Edwards Deming's [21] 14 points on quality control called for reducing the variability in the production process of goods and services. Therefore, having a more streamlined approach to the management of critically ill patients would make sense for better outcomes. Hospitals have little financial incentive to incorporate technology assessment into their quality assessment processes. For example, the Swan-Ganz catheter is a monitoring device long used in the care of patients. However, no solid data exist to claim that its use is cost-effective and that it can make a definite improvement in quality outcomes [18, 22, 23].

Should standardized means exist for assessing technology to provide for cost-effective and beneficial care? Would developing these types of approaches lead to rationing of critical care services? The concept of rationing has some ethical concerns that will be addressed. However, some people, such as Kindig [3], point out that we already are rationing health care, by virtue of the fact we already have the problem of 46 million people without health insurance.

In the United States, the Society of Critical Care Medicine has made suggestions for uniform assessment of monitoring devices [24]. In particular, working with industry in a constructive fashion to make improvements can be done without any “conflicts of interest.” Feedback to the clinician at the bedside is important. The Society of Critical Care Medicine has suggested a closed-loop type of system [25] in which information could be provided to the clinician on outcomes of trials before implementation of the technology.

Solutions to technological costs will not be easy. Managed care competition is not able to control rising health care costs, provide care for the majority of the population, and guarantee quality health care outcomes. Schwartz [26] has suggested that health care technology will continue to increase costs. The previous factors as described by Newhouse [5] will continue to fuel health care inflation.

Sibbald et al. [27] has called for assessment of which patients would benefit from high-cost technology, use of technology assessment for total quality improvement, and evaluation of new technologies by health care funding agencies. This makes sense in that the US Food and Drug Administration (FDA) evaluates all drugs before permitting them to go out to market. Should not we do the same with technology? Safety and efficacy of drugs has been the FDA's greatest concern. If the FDA were also to perform technology assessment of certain key devices, the costs, effectiveness, and utilization of new medical technologies may be better understood. Whether the FDA should be responsible for this is open to debate. Recently this has come into question with regard to implantable cardioverter-defibrillators and their safety concerns as addressed by the FDA [28].

This question begs investigation with regard to information systems. In particular, the Leapfrog Group, in pushing forward its standards for higher quality of care, has made recommendations for electronic information systems and Computerized Physician Order Entry as ways to improve quality of care and reduce errors [30]. However, there is no strong evidence that this will make things better. There are no randomized prospective trials to provide the evidence to validate these recommendations by the Leapfrog Group. And there is no strong evidence that this would lead to better decision making with regard to utilization of ICU resources for critically ill patients who will benefit the most.

## 4. Ethics in the ICU

Utilization of ethical principles is a common occurrence in the ICU. The ICU presents an environment in which patients and their families tend to have a great deal of stress. In an ICU setting, patients often undergo resuscitation [31] and often have numerous medical interventions, including life support with ventilators for failed respiratory function, dialysis for failed kidney function, treatment of hemodynamically unstable shock states with vasopressors, treatment of sepsis with antibiotics, organ transplantation, and nutritional support with enteral feeds or total parenteral nutrition. Other extreme modalities have included placement of heart-assist devices and use of extracorporeal membrane oxygenation. Additionally, it is often necessary to have more intensive nursing available. All of this is incorporated into the high cost of critical care in the ICU.

Scoring systems such as APACHE have been instituted to allow for some assessment of risk adjustment [32]. However, not infrequently, a point is reached at which intensive care may become futile [33]. Four principles of ethics should be assessed by all providers that care for patients in the ICU, as outlined by Beauchamp and Childress [29] (Box 3). These 4 principles are autonomy, beneficence, nonmalfeasance, and justice.

Health care is beginning to incorporate the principle of autonomy (“self rule”), meaning that the patient has the right to control decision making over his or her own health needs. This became more evident in the 60s and 70s with some of the principles of the “Great Society” and the institution of Medicare. This concept is encountered daily in the ICU. For example, do patients want life-sustaining support from certain medical machines that may prolong their lives but may not offer a final cure? The fundamental question is “What does the patient want?” The answer often is not easy. The patient may be incapacitated, and decision making may fall to another person, as in the case of Terri Schiavo [34]. In that case, the decision-making capacity was given to Ms. Schiavo's husband. Without a clear advance directive, arguments ensued between the husband and Ms. Schiavo's family as to her wishes for life-sustaining treatment. This illustrates that every person should have an advance directive stating her or his wishes in potential end-of-life situations.

What is autonomy? I believe that autonomy involves more than refusal of care. Autonomy does not mean that patients demand extraordinary care that will not benefit them [33]. For example, imagine a 92-year-old with end-stage chronic obstructive pulmonary disease is nearing the end of his life on the ventilator. The daughter reads that “lung transplants” are quite common and demands that her father be evaluated. It would clearly be inappropriate to offer an organ transplant to someone who would not benefit from it. This illustrates the “asymmetry of information” mentioned previously. The patient clearly may not be able to fully understand his options given all of the appropriate information.

The next principle of ethics is “beneficence,” defined by Beauchamp and Childress [29] as doing “good” for the sick. Surely, if someone has pneumonia, no one would argue against instituting therapy immediately. The question comes when appropriate treatment does not elicit a response and all options are exhausted: What are our next options? This situation is common in end-of-life decisions.

The next guiding principle is nonmalfeasance, “to cause no harm” [29]. This becomes a difficult situation at a point in care where no further benefit is given and suffering may just be prolonged. An example again is the 92-year-old with terminal chronic obstructive pulmonary disease who cannot be liberated from the ventilator. One could keep supporting the patient on the ventilator, but to what end? Here, the principle changes from beneficence to nonmalfeasance in that withdrawal of life support should then be considered.

Finally, the principle of justice is defined as doing the greater good for everyone concerned [29]. Generally, between the provider and the patient, this principle is often not addressed. This may result from our American principle of observing the rights of the individual. But is it “justice,” for example, for a bedridden 90-year-old to receive dialysis while a poor 6-year-old cannot afford eyeglasses to see in school? These are questions for Americans to consider.

The Clinton health plan offered some new ideas for health care coverage in America. In my opinion, it failed in part because of the heterogeneity of American society. Waymack [35] states that the Clinton health plan had contradictory elements from “respecting equal access versus individual responsibility and choice versus health care being a moral right or privilege versus health care being a social good or a free market commodity.” These issues, such as possible allocation of health care resources and dollars and possible rationing, should be dealt with at a higher administrative level, such as the government. Information systems will have a major role in making some of these decisions.

## 5. Rationing, Ethics, and the ICU

The word *ration* as a noun means a “fixed allotment to be dealt out or distributed”; ration can also be a verb [36]. In the context of health care, *rationing* implies that resources will be denied or redistributed to certain patients. This issue was addressed at the 2005 annual meeting of the Society of Critical Care Medicine [37]. Dr. Mitchell Levy, along with the society's ethics task force, defined *rationing* as “the allocation of health care resources in the face of limited availability, which necessarily means that beneficial interventions are withheld from some individuals” [38]. Dr. Levy states that we already are rationing critical care health resources without even realizing.

According to Dr. Levy, we ration on a daily basis by “allocating our time to certain patients, triaging of beds, withholding diagnostic procedures in patients who are unlikely to benefit, how aggressively to treat certain patients with ‘end of life' diseases, and so forth” [38]. Rationing, in a sense, might be the ethical thing to do.

In 1994, a survey was sent out to critical care professionals to test the attitudes of providers regarding rationing [39]. The results of the study showed that quality of life of the patient, the probability of surviving, whether the acute illness could be reversed or not, and the nature of a chronic disease all played a role in the provider's mind as to whether resources could be distributed fairly. Economic background of the patient did not play a role in this decision. It seems logical that it would not make sense to waste resources on care for a patient with a terminal disease if the outcome would have no benefit. This especially seems prudent considering that resources will be limited in the future. But how can a physician act wisely with good clinical and ethical judgment? Prognostic scoring systems have been shown to be useful in some cases, but they have not been universally adopted [40]. This may call for some agreed-upon scoring system with use of an electronic means for determining utilization of resources and for decision making. This would indeed have to be a system that is not only used by physicians but understood by patients and their families.

In Canada, authors such as Cook and Giacomini [41] have discussed rationing. In their article, they point out that health care resources are fixed but the demand for health care is not. At the patient care level, rationing could occur with respect to technology, treatment, ICU bed days, and hospital bed days. This could come into gross conflict with patient autonomy. Some patients and families demand extraordinary resources despite indications of poor outcome. The question of whether information resources and avenues of care would also be rationed was not specifically addressed. But decisions are not translated down to the bedside to address the issue. Resources will remain fixed and may even decrease, but patient demand will continue to grow over time [1]. Information resources may better bridge the gap or lead to more conflict with regard to allocation of expensive resources in critical care.

One reasonable solution to rationing would be to identify which patients would not benefit from critical care and then transfer those patients to a more palliative situation. This makes sense given that ICU resources would be wasted on someone who would not have a good outcome. However, there is evidence that this may not be the case. Luce and Rubenfeld [42] pointed out that this would not produce great cost savings because (1) most hospital costs are fixed and would not be influenced by decreases in ICU length of stay; (2) current prognostic systems lack the necessary specificity to predict death and therefore could not predict which patients would be the costliest; and (3) only a minimum number of patients have dismal prognosis and it would not substantially reduce costs by withholding ICU care from them.

Luce and Rubenfeld [42] may have some valid arguments; the 3 points listed above are correct to a degree. However, I believe that their observations are not based on broad data. Costs are either fixed or variable, but it is not clear how variable costs can play a role in allocation of resources for critical care. Prognostic scoring systems provide different answers in different environments. Finally, the severity of illness can vary from different institutions.

When patients are asked about code status or whether they would want heroic treatments, for the most part they are never asked about a situation that may involve a terminal state. Questions like this should be addressed to all patients with serious chronic illnesses or in terminal states. This could be an excellent use of electronic records whereby information such as a patient's wishes for heroic measures are documented ahead of time in an electronic form. Patients could be asked about their preferences when they are not critically ill and have their responses documented. A needless admission to the ICU could be avoided. The patient's autonomy could be respected, no harm would be done, the best care could be offered, and societal needs could be met. Electronic means of documentation via personal health record would be very useful. However, a patient's rights to privacy and nontampering of the record to ensure security would be highly necessary [43].

Kindig [3] writes about rationing on a population-health basis. He states that “if health care is a public or private good and therefore we ration on the ability to pay” [3]. In essence this means that a patient who cannot afford health insurance cannot have access to health care. Kindig views this as a form of rationing. Kindig makes a strong argument that we can and should alter “health adjusted life expectancy” to the maximum amount of dollar input. In other words, dollars spent on quality of life that do not influence outcome should not be spent.

Cost-effectiveness of health care should be determined. Kindig points out that numerous tables are available that list the cost-effectiveness of certain high-priced interventions. In terms of rationing, we should limit treatments that are expensive with little beneficial outcome. Certain treatments have high prices but often result in nonbeneficial outcomes, or negative outcomes can result from iatrogenic complications [3].

Kindig points out that factors other than health care can lead to potentially better outcomes. Higher income, higher education attainment, and smoking cessation all are factors that can lead to a healthier population [3]. The state of Oregon was the first to develop a plan for rationing based on cost-effectiveness [3]. This plan has had some criticisms, but it is the first of its nature to address allocation of health resources that are very expensive and whether or not they will be truly effective. There have been some misconceptions about the Oregon plan. However, Oberlander et al. [44] have addressed some of these issues.

1. There has been no widespread rationing of resources within the state of Oregon.
2. To establish a limit on the amount of services is more difficult than expected.
3. Oregon's lists of priority setting has not produced savings.
4. No other state has copied the Oregon model. There is a reluctance to add any concept that may result in denial of services.

Other means to address the concept of rationing would be necessary. Eddy [45] proposed a rationing structure that seemed to make sense.

1. Resources are limited.
2. Help patients understand the consequences of limits and the need to be fair.
3. Change to thinking as the “most benefit per dollar.”
4. Focus on populations rather than individuals. Resources wasted on ineffective treatments for one individual come from other patients.
5. Measures of quality need to support the strategy for increasing outcomes while decreasing costs.

This above assessment follows more the “justice” approach than the “autonomous” approach. Certain patients may be reluctant to accept this approach. However society must realize the limited resources available. Electronic information systems can play a key role in this not only for provider but also for patient.

## 6. Information Systems: What is Its Role with Regard to Ethics, Rationing, and Technology Assessment?

Electronic information systems are beginning to play a key role in health care management. Bates and Gawande [46] discussed ways that the electronic record will be useful in the ICU. They state that identifying adverse events and errors, facilitating a more rapid response after an adverse event has occurred, and tracking and providing feedback on adverse events can lead to better outcomes. Electronic data systems in the ICU can improve communications, provide access to information, help with medication safety, and assist with calculation, monitoring, decision support, rapid response, and tracking of events [46].

The notion of “rationing” or only allocating resources to some part of the populace can be viewed in different forms. It can be done actively or, as we are doing now, passively. Will information systems help the ICU physician provide the right care to those that need it?

Information systems can be associated with ethical issues. Goodman [47] states that new technology can seduce us into some expensive forms of care such as organ transplantation, dialysis, life support, and other forms that carry high price tags with little data on cost-effectiveness. How do we manage care for this critically ill group of patients with shrinking financial resources in the future? It is apparent that coordinating ethical decisions based on the best information from evidence-based medicine will be the wave of the future. Here is where informatics will play a major role in decision making at the bedside.

According to Goodman [47] ethical issues of patient information include confidentiality, standards for errors, health information networks between various hospitals, consultation via telemedicine, advance directives, monitoring, case management, informed consent, and organ transplant registries. Advance directives, for example, are often not addressed until the patient has been in the ICU for several days. When the subject is discussed, families often state that the patient “would not have wanted all of this heroic treatment.” Addressing this issue beforehand with a patient would be the sensible thing to do. According to Jacobs and Noseworthy [48], ICU costs contribute significantly to overall expenditures. Avoiding services that are expensive but afford no meaningful quality outcomes may help to contain some of these expenditures without resorting to outright denial of services.

As stated before, it would seem intuitive to identify patients that may not benefit from critical care. Luce and Rubenfeld [42] do not believe that this is feasible because these patients only constitute a minority of ICU admissions. However, it can be argued that demand for ICU resources is increasing, with the baby boomer generation now coming into the retirement years [1]. It seems that there is nothing wrong with documentation of a patient's wishes for life support ahead of time. This may result in more savings in health care resources.

With the assumption that health care information in an electronic form could provide practitioners with the best information for clinical decision making, certain ethical issues must be considered. In particular, could decision making coupled with information on technology assessment of various therapies lead to outcomes without rationing? With regard to electronic records, Kaplan [49] cites shortcomings in record integrity, decision making, and privacy and security as ethical concerns that need to be dealt with. Will information that is transmitted within the hospital, within networks, or outside of networks maintain its integrity and confidentiality to benefit the patient and help the clinician make the correct decisions?

In 2003, Sibbald [50] described the outcome of a workshop with regard to technology assessment in critical care. The several outcomes presented included questions about how the evaluation process was accomplished and what evidence is required to adequately evaluate a device for use in the ICU. Other findings were that medical dissemination generally occurs before there is adequate evaluation, and decision making about purchasing certain forms of technology occurs in a rather haphazard manner, without evaluation by the people that use it [50]. Utilization of technologies without proper evaluation leads to higher cost, which places a strain on the system when coupled with the higher demand for ICU services. Consequently, we must decide who gets what technology for treatment and to what end. Hence, this is a form of rationing. If we keep on this path, are we doing ethical justification to our patients?

Widespread consensus exists that the use of electronic records within hospitals could lead to better outcomes [51]. However, recent surveys have shown that only 1.5% of US hospitals have a comprehensive electronic records system for all units of the hospital [51]. Capital requirements and maintenance costs were cited as the major barriers. If indeed this is a necessary element to control our health care costs, improve quality outcomes, and assure better utilization of resources, then it seems prudent that the initial investment be made on a wide scale.

Examples of technology in the ICU include noninvasive positive pressure ventilation, monitoring, total parenteral nutrition, and many others. Sinuff and Cook [52] describe a process of clinical evaluation of noninvasive positive pressure ventilation for support of patients with exacerbations of chronic obstructive pulmonary disease. Their 11-point approach is as follows. (1) What is the efficacy of the technology? (2) Has the technology been evaluated in randomized trials? (3) Does technology improve outcomes with best practices? (4) What are the treatment effects? (5) Is there a range of use of the technology? (6) Can I apply the technology in my practice? (7) Can I expect similar clinical benefit? (8) Were important outcomes considered? (9) Are outcomes dependent on protocols, operators, or hospital location? (10) Has the health care resource of the technology been evaluated (i.e., the manufacturer)? And (11) How much time has been utilized by the health care practitioners that use the technology [52]?

This seems like a long and lengthy process, but it is necessary given the realities of the economic future of critical care medicine [27]. Information systems will have to play a role in uniting the pieces of this complicated puzzle. With proper information, scoring systems for outcomes, a priori documentation of the patient's wishes for heroic services, technology assessment, and ethical judgment, better decisions could be made about redirecting critical care resources. Is this a form of rationing? In the extreme sense of the word, I think not. No patient would be denied care they need or from which they could benefit. However, administration of futile care is costly, unnecessary, and ethically questionable [33]. Physicians should not be obligated to provide futile care. However, families and patients should feel very comfortable about their decisions made based on information from the providers.

An example of possible futile care is putting some patients in their 80s on chronic dialysis, regardless of the situation of the patient. Often, families think of this as a benign support tool, but it can have many complications and is a very expensive resource. Would not it make sense to have data stating whether this patient would benefit or not and whether the treatment is cost-effective? Electronic information systems could offer patients more information to see why certain decisions need to be made. With this, rationing, in the cruel sense of what some people may perceive it to be, may not have to be used.

It is most ethical to render the best care possible to critically ill patients. Issues to address include (1) foregoing life-sustaining technology for those who will not benefit; (2) ensuring patient autonomy; and (3) finally allocating resources in the ICU on the basis of best standards of technology assessment [3, 7]. Patients need to be better educated with regard to self-determination and outcome data on their individual disease. This will hopefully make for better decision-making processes when dealing with the allocation of resources.

## 7. Conclusions

In conclusion, several points are to be made:

1. Critical care services continue to remain a high-cost specialty due to demand for services and high-priced technology.
2. Services for health care are being rationed today as evidenced by the fact that 46 million Americans do not have health care coverage. The number of people that have no access to ICU services remains unknown.
3. Risk adjustment systems such as APACHE have been studied but are yet to provide definite means to help allocate services in a way that is most beneficial.
4. Real-time rationing will become a reality with withholding of services unless costs can be controlled.
5. Electronic health records with decision support can help in deciding how services can best benefit patients. This should incorporate technology assessment of some of the most expensive items in health care.
6. Critical care must continue to work toward a scoring system for risk adjustment that is fair, equitable, and ethical.
7. With today's critical care, it is very difficult to do no harm, do what is right, but yet be fair and equitable to all. Electronic records will hopefully help with safety and error reduction.
8. Electronic records should also document living wills and advance directives with regard to extraordinary care. This will hopefully avoid use of unnecessary services for people who will not benefit.
9. Electronic records should ensure confidentiality, accuracy, and security. This is particularly necessary when considering possible allocation of some of these expensive services.

## Figures and Tables

**Box 1:**
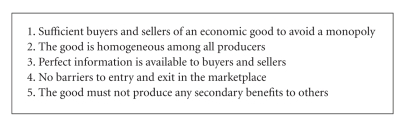
Necessary factors for free market competition [4].

**Box 2:**
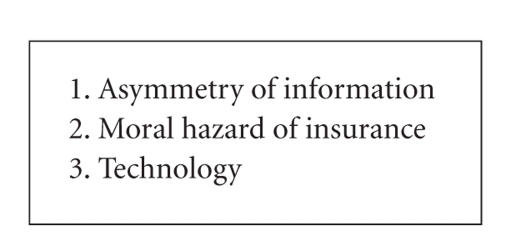
Causes of health care inflation [8].

**Box 3:**
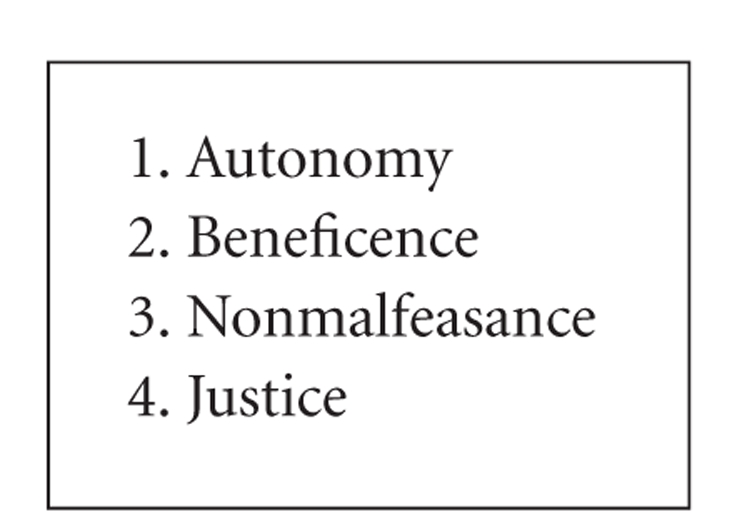
Principles of ethics [29].
